# Maternal Residence Near Agricultural Pesticide Applications and Autism Spectrum Disorders among Children in the California Central Valley

**DOI:** 10.1289/ehp.10168

**Published:** 2007-07-30

**Authors:** Eric M. Roberts, Paul B. English, Judith K. Grether, Gayle C. Windham, Lucia Somberg, Craig Wolff

**Affiliations:** 1 Public Health Institute, Oakland, California, USA; 2 California Department of Health Services, Richmond, California, USA; 3 School of Public Health, University of California, Berkeley, California, USA

**Keywords:** autism spectrum disorders, health surveillance, methods, organochlorines, pesticides

## Abstract

**Background:**

Ambient levels of pesticides (“pesticide drift”) are detectable at residences near agricultural field sites.

**Objective:**

Our goal was to evaluate the hypothesis that maternal residence near agricultural pesticide applications during key periods of gestation could be associated with the development of autism spectrum disorders (ASD) in children.

**Methods:**

We identified 465 children with ASD born during 1996–1998 using the California Department of Developmental Services electronic files, and matched them by maternal date of last menstrual period to 6,975 live-born, normal-birth-weight, term infants as controls. We determined proximity to pesticide applications using California Department of Pesticide Regulation records refined using Department of Water Resources land use polygons. A staged analytic design applying *a priori* criteria to the results of conditional logistic regressions was employed to exclude associations likely due to multiple testing error.

**Results:**

Of 249 unique hypotheses, four that described organochlorine pesticide applications—specifically those of dicofol and endosulfan—occurring during the period immediately before and concurrent with central nervous system embryogenesis (clinical weeks 1 through 8) met *a priori* criteria and were unlikely to be a result of multiple testing. Multivariate *a posteriori* models comparing children of mothers living within 500 m of field sites with the highest nonzero quartile of organochlorine poundage to those with mothers not living near field sites suggested an odds ratio for ASD of 6.1 (95% confidence interval, 2.4–15.3). ASD risk increased with the poundage of organochlorine applied and decreased with distance from field sites.

**Conclusions:**

The association between residential proximity to organochlorine pesticide applications during gestation and ASD among children should be further studied.

“Autism” refers to a set of neurodevelopmental disorders that are characterized by impaired social interaction, restricted communication, and repetitive, stereotypic behaviors. The number of children reported as having autism spectrum disorders (ASD) has risen dramatically since the early 1990s. In the United States, some of this increase is attributable to changes in diagnosis and reporting, although this pattern is not uniform across all states (Shattuck 2005). Symptoms of classic autism do not typically become evident until early childhood, but current evidence is consistent with a pathogenic process originating during fetal development (Arndt et al. 2005; Hertz-Picciotto et al. 2006; Kemper and Bauman 1998; Nelson et al. 2001).

Many of the hypotheses regarding ASD pathogenesis involve a functional deficit caused by alterations to specific brain structures occurring *in utero* during defined temporal windows of vulnerability (Polleux and Lauder 2004). The lesions in question might result from genetic factors, environmental insults, or a combination of the two. A variety of lesions could give rise to a “final common pathway” to autism; ASD as currently defined may well include multiple disorders that have not yet been successfully differentiated.

A large number of widely used agricultural pesticides have known neurologic effects (Weiss et al. 2004), raising the possibility that gestational exposure to these compounds could play an etiologic role in ASD and related neurodevelopmental disorders. Most compounds are prone to “drift,” and detectable levels in air samples are often measurable at locations beyond the site of application for extended periods afterwards (Kegley et al. 2003; Lee et al. 2002). Elevated levels of agricultural pesticides in household dust and their metabolites in urine have been associated with residential proximity to treated fields (Loewenherz et al. 1997; Lu et al. 2000; Simcox et al. 1995).

Studies of pediatric diseases and their associations with residential proximity or parental occupational exposure to pesticides have been accumulating, most notably for cancer (Birnbaum and Fenton 2002; Daniels et al. 1997; Feychting et al. 2001; Flower et al. 2004; Linet et al. 2003; Meinert et al. 2000; Olshan et al. 2000; Reynolds et al. 2002; Robison et al. 1995; Shannon 1998; Zahm and Devesa 1995) and, more recently, neurodevelopmental delay (Grandjean et al. 2005). Many environmental toxicants are conveyed transplacentally, and the blood–brain barrier remains relatively permeable to many of these compounds until well into the first year of life (Andersen et al. 2000). In general, experimental and epidemiologic evidence regarding pesticides and pediatric neurodevelopment is strikingly lacking, despite considerable knowledge about pesticide toxicity (particularly neurotoxicity) (Kamel and Hoppin 2004; Weiss et al. 2004).

We evaluated a series of hypotheses regarding an association between *in utero* residential “exposure” to specific agricultural pesticides (that is, maternal residence in close proximity to sites of application) and the development of ASD by linking existing databases using a retrospective case–control design. This study was conducted as part of a demonstration project of the utility of environmental public health tracking, an initiative funded by the Centers for Disease Control and Prevention (McGeehin et al. 2004) to generate clues for further etiologic study.

## Materials and Methods

All data assembly and sampling protocols were approved by the California Department of Health Services Committee for the Protection of Human Subjects before the initiation of activities.

### Study subjects

The study population included 269,746 singletons born between 1 January 1996 and 31 December 1998 to mothers residing in the 19 counties included in the Sacramento River Valley and San Joaquin River Valley air basins of California (known together as the Central Valley) (California Center for Health Statistics, Sacramento: Birth Statistical Master Files, unpublished data). Excluded from the study population were infant deaths and multiple births. Modestly elevated ASD risk has been noted among children born preterm (Larsson et al. 2005); in this exploratory study, we focused on ASD risk not mediated by this phenomenon and excluded those born at < 37 weeks gestation or weighing < 2,500 g. Children with ASD were identified from this study population through electronic files of the California Department of Developmental Services (DDS) (California DDS, Sacramento, CA, Client Development Evaluation Reports, unpublished data), which operates a statewide system of regional centers and developmental centers that coordinate voluntary services for persons with autism, mental retardation, and other developmental disabilities. Children reported by DDS at any age as receiving services for autism or with an ASD diagnostic code (e.g., *Diagnostic and Statistical Manual of Mental Disorders*, 4th ed., code 299.80) (American Psychiatric Association 2000) were included in the case group.

DDS eligibility is determined and services are provided without regard to citizenship or financial status. Although the system is used widely across different socioeconomic levels and racial and ethnic groups, disparities in use may exist. Referrals come from pediatricians, other clinicians, the educational system, family members, and friends. DDS creates an archive file of client development evaluation reports (CDER) completed at the regional centers and developmental centers. A CDER is submitted when an individual has a diagnosed developmental disability and has met the eligibility requirements for active status in the DDS system. Children who qualify for services for conditions other than autism but who have comorbid ASD may have a diagnosis of ASD coded on their CDER under “Mental Disorder.” Children with milder forms of developmental disabilities, including Asperger’s Syndrome and Pervasive Developmental Disability–Not Otherwise Specified (PDD-NOS), may not meet eligibility requirements for active status. The CDER is updated periodically as client status changes.

### Linkage of case subjects to birth records

To identify DDS clients who were resident births and to obtain street address at birth and other demographic variables for analysis, DDS electronic files were linked to live birth vital records by staff of the California Center for Autism and Developmental Disabilities Research and Epidemiology (Richmond, CA). Matching algorithms were based on identifying variables including child’s first name, last name, date of birth, sex, and mother’s first name, last name, and date of birth. We estimate an incorrect matching rate of < 0.2% using these procedures (Grether J, unpublished data).

### Control subjects

For each case, we selected 15 control births from the study population from among full-term, normal-weight live births who were not identified as cases using an incidence density sampling design with date of last menstrual period (LMP) as the time variable. This procedure permitted control for the time-varying prevalence of exposure that could, uncontrolled, result in confounding, and maximized the information obtained from the cohort for estimation of disease rate parameters. For the small number of records for which gestational age was missing, we imputed this number based on the date of birth and LMP. Records for which the (recorded or imputed) gestational age was incompatible with the recorded birth weight using an established algorithm (Alexander et al. 1996) were excluded before sample selection.

### Regional center as a covariate

DDS regional center (RC) catchment areas are geographically defined, and services are provided based on residence address. Six RCs serve the 19 counties included in this study; because of migration between birth and age of diagnosis, 19 RCs contributed diagnoses to cases in the study population. Statewide guidelines are provided for eligibility determination and provision of services, but RCs have flexibility in application of guidelines. To adjust for differences among RCs, we included a variable for cases indicating RC of enrollment when eligibility based on autism was established. For control subjects, RC assignments were simulated under the assumption that migratory patterns during the first few years of life would be identical between case and control populations. For each RC, we calculated out-and in-migration between birth and CDER diagnosis date for ASD cases, and then randomly selected an identical proportion of controls born in each RC catchment area and reassigned them accordingly. Later when choices had been made regarding the analytic model, we repeated the random assignment 100 times to assess the sensitivity of our findings to this process.

### Pesticide data

We obtained records from the California Department of Pesticide Regulation (DPR 2000) describing agricultural pesticide applications within the study area occurring after 1 January 1995 (for the 4 years after this date, the total number of applications was 6,710,727). These data are submitted to DPR by county agriculture commissioners and are spatially referenced to public land survey sections (PLSS); we conducted cleaning and correction algorithms following the protocol of Gunier et al. (2001). Following the method of Rull and Ritz (2003), we spatially refined these data through the overlay of matched land-use survey field polygons provided by the California Department of Water Resources (DWR 2005). Briefly, we matched each DPR record to the land-use survey conducted closest in time to the application date (DWR surveys are conducted roughly every 5–7 years in each California county). Matching is based on location and crop type as specified in both the DPR and DWR records, with frequently rotated crops grouped together in a single category. Of the total applications recorded by the DPR spanning 1995–1998, 73.4% were successfully linked based on specific crop identifiers, whereas an additional 18.0% were linked under the “frequently rotated” category. For the remaining 8.5% of applications, no field polygon in the specified PLSS grid was identified with the appropriate crop identifier, so no spatial refinement was possible (percentages do not sum to 100 due to rounding).

### Data linkage

Exposure assignment incorporated both spatial and temporal dimensions. Residence addresses at time of birth were standardized and verified using ZP4 (Semaphore Corporation, Pismo Beach, CA) and subsequently geocoded using ArcGIS version 9.0 (ESRI, Redlands, CA). Geocoded address coordinates were taken from the first successful match of the following four street centerline data sets (in order): Geographic Data Technology (GDT) Dynamap/2000 version 13 (TeleAtlas, Lebanon, NH), Navigation Technologies NAVSTREETS (NAVTEQ, Chicago, IL), TeleAtlas MultiNet streets (TeleAtlas), and the U.S. Census Bureau TIGER 2000 system (Washington, DC). For each street centerline data set, residences were geocoded by matching to street address attributes (e.g., prefix, number, name) and the ZIP code, or, if the ZIP code was unsuccessful, the city name.

We determined temporal proximity by comparing dates of applications recorded in the DPR data set (which are believed to be accurate within a few days) to the stage of gestation (quantified as the number of days postfertilization) determined from LMP. Fertilization was assumed to occur 14 days following the LMP date and labeled day zero. LMP is therefore day –14, and the expected delivery date for a full-term pregnancy day 266.

To assign exposure, we developed a custom Java (Sun Microsystems, Santa Clara, CA) application using the ArcSDE Java Application Program Interface version 9.0 (ESRI, Redlands, CA) and the GeoTools Java GIS Toolkit, version 2.0 (open source, http://geotools.codehaus.org/). We calculated the sums that combined the numbers of pounds of pesticides occurring during any temporal window (defined below) within the specified radius of a geocoded point, intersecting DWR land-use or PLSS polygons with the buffer, and assuming homogeneous distribution of pesticides within each of these polygons.

### Analytic strategy

The infrastructure developed for this project allowed us to simultaneously test large numbers of specific hypotheses. To avoid the pitfalls associated with multiple statistical comparisons, we constructed a multistage analytic strategy. First we selected pesticide compounds based on plausibility of biological connection to autism, physical characteristics, and community concerns. Then we operationalized the hypotheses of association between exposure and outcome based on known embryologic phenomena. We then conducted *a priori* data analysis with primary attention to the effects of multiple testing, followed by *a posteriori* data analysis for refinement of hypotheses and guidance of future work.

#### Selection of pesticide compounds

Input was obtained through a series of participatory meetings with representatives of community-based, local governmental, and nongovernmental organizations. Two complimentary and overlapping sets of criteria were developed to select pesticides of interest: *a*) compounds causing substantial community concern, and *b*) compounds most likely to have spatially and temporally resolvable health effects based on their toxicologic and physical properties. Community concerns included pesticides accounting for particularly large proportions of total agricultural applications in the state; pesticides associated with well-known involuntary exposures due to incidents of community poisonings; and fumigant pesticides, which are used in particularly large quantities during a single application. To address the second set of criteria, we assembled a list of 54 high-use pesticides known to be neurotoxicants, reproductive toxicants, developmental toxicants, and/or endocrine disruptors (Green Media Toolshed 2006). Following previously developed protocols (Bennett et al. 1999; MacLeod et al. 2004), we ranked compounds from the list by a local exposure index, which is the environmental persistence weighted by the fraction of deposition expected to occur near to the application site. The resulting individual compounds and groups of compounds are shown in Appendix 1.

#### Operationalization of hypotheses

We operationalized each single hypothesis as any unique combination of *a*) pesticide compounds or groups of compounds, *b*) maternal residential distance from application site, and *c*) temporal period during pregnancy. Spatial parameters were based on the assumption that substantial population exposure due to pesticide drift was unlikely at distances > 1,000 m and may be restricted to distances smaller than a few hundred meters (Kegley et al. 2003). We tested hypotheses using distances of 250, 500, and 750 m for both individual and grouped compounds. For grouped compounds, we additionally tested hypotheses using a 1,000-m radius.

Temporal parameters were chosen to reflect the hypotheses that the periods immediately before and during central nervous system (CNS) embryogenesis, neural tube closure, and entire gestation could represent critical windows for exposure. We defined these respective periods as follows: CNS: days –7 through 49; neural tube: days –4 through 24; and gestation: day –14 through date of birth.

#### *A priori* analysis

For each parameter combination, we considered only instances for which a minimum of five case or control subjects per quartile of nonzero exposure were available. We imposed the following standards for consideration of any associations between pesticide exposures and ASD as significant.

The fourth nonzero quartile coefficient must be significantly greater than zero, with *p* ≤ α*_adj_* using the Holm algorithm (Aickin and Gensler 1996) and incorporating the formula by Dunn-Sidák (Nichols and Hayasaka 2003). Under this algorithm, the adjusted alpha becomes

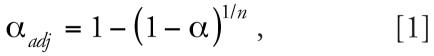



where α is 0.05 and *n* is the rank (1,2,3,…) in *p*-value, beginning with the smallest.

Further, we graphically depicted the distribution of *p*-values, allowing the data themselves to suggest a logical cut point for the exclusion of associations likely to be attributable to multiple testing.

For screening purposes, we employed a conditional logistic regression model (using LMP date to define strata for case–control matching) that controlled for maternal race/ethnicity, maternal education (classified as elementary, some high school, high school graduate, some college, and college graduate), and RC of diagnosis (actual for cases and imputed for controls). For exposure, the reference category was “none,” with separate coefficients for each of the four nonzero quartiles of pesticides, in pounds. All variables in sthe model were considered categorical, meaning that no linearity of effects was assumed.

#### *A posteriori* analysis

Further analysis was restricted to *a priori* combinations of parameters demonstrating significant associations with risk of ASD using the above criteria. We adjusted temporal parameters by making them an 8-week moving window extending from 300 days before to 300 days after the estimated date of conception. This yielded an “optimal” parameter combination that we could use to assess model sensitivity and to characterize the dose–response relationship between pesticide applications and ASD risk. For the latter, we estimated a LOESS function of pesticide applications (in pounds) following the methods recommended by Figueiras and Cadarso-Suárez (2001). Optimal span for the LOESS function was chosen as that which yielded the minimum value for Akaike’s Information Criterion (Akaike 1973). The dose–response analysis was conducted using the *gam* package developed by Hastie (2006) for use in the *R* programming language version 2.3.1 (R Development Core Team 2006); all other analysis was conducted using SAS version 9.2 (SAS Institute Inc., Cary, NC).

## Results

Of the original 269,746 singleton births, we were able to geocode 94.6%, with only negligible differences in this rate between case and control subgroups. A further 4.6% of these records were excluded because estimated gestational age was incompatible with birth weight. From the remaining births, we identified 465 ASD cases plus 6,975 matched controls. ASD cases were 85.2% male; for controls this proportion was 51.4% (further information on demographic characteristics is shown in Table 1). Eight cases and 100 controls had missing data for at least one covariate of interest; in nearly all instances this covariate was maternal education. For each regression model, only subjects with complete information for all necessary covariates were included.

### A priori *analysis.*

A total of 249 combinations of compounds, buffer radii, and temporal periods met the requirement of five exposed cases and controls per cell. The coefficients comparing the fourth nonzero quartile of exposure to the reference category are presented for the eight combinations where the *p*-value was < 0.05 (the unadjusted α) in Table 2.

Regardless of buffer radius, all fourth nonzero quartile coefficients meeting our numeric criterion for significance adjusted for multiple testing were for the category of organochlorine pesticides with applications occurring during the CNS period; furthermore, only regressions with this compound–temporal period combination yielded *p*-values that met this criterion. Generally, these coefficients had *p*-values an order of magnitude smaller than those for the next most significant coefficients.

**Figure 1:**
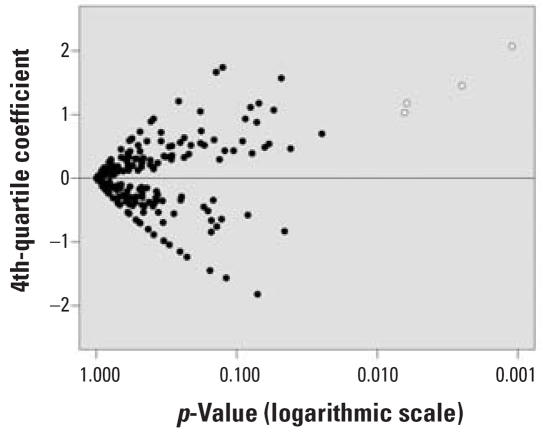
Coefficients for ASD risk comparing the fourth nonzero quartile of exposure to no exposure among children born in selected California counties, 1996–1998. Only coefficients for which a minimum of 20 subjects had nonzero exposure are shown. Model controls for maternal education, maternal race/ethnicity, and RC of diagnosis (imputed for controls). Open circles represent coefficients for organochlorine pesticides applied during the CNS period; closed circles represent all others.

The *p*-values and fourth nonzero quartile coefficients are plotted in Figure 1. As expected because of multiple testing, most of these coefficients are randomly distributed around zero, with a few having *p*-values close to 0.05. The coefficients for organochlorine exposure during the CNS period, in contrast, have *p*-values substantially smaller than their nearest neighbors on the graph and are consistently positive.

Organochlorine pesticides were found to be associated with ASD regardless of the buffer radius used. The effect becomes monotonically smaller as the radius gets larger; when the buffer radius is extended to 1,750 m, the fourth nonzero quartile odds ratio (OR) finally becomes nonsignificant (*p* > 0.05; data not shown). For the *a posteriori* analysis, we selected the radius of 500 m, which was the smallest for which there was at least one case for each exposure category.

### A posteriori *analysis.*

**Figure 2:**
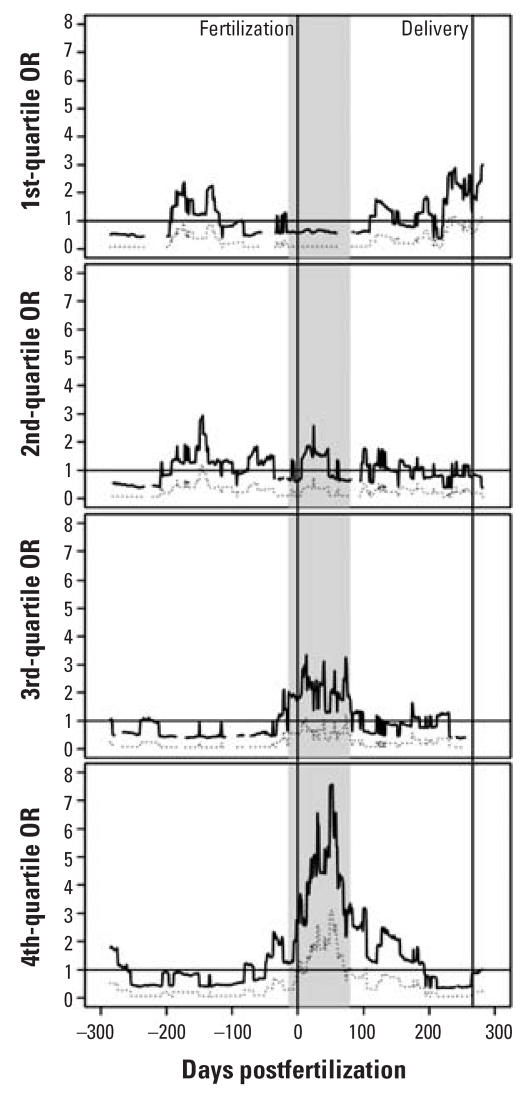
ORs (solid lines) and lower 95% confidence limits (dotted lines) for ASD comparing nonzero quartiles of organochlorine pesticide applications within 500 m to no applications for overlapping 8-week temporal windows. Models control for maternal education, maternal race/ ethnicity, and RC of diagnosis (imputed for controls). *x*-Axis is the date in the center of each temporal window relative to fertilization date; shading indicates clinical first trimester; gaps indicate no ASD cases occurred for that category.

Only organochlorine compounds met the criteria for inclusion in *a posteriori* analyses Using a 500-m radius around residential locations, we allowed the 8-week temporal window to be centered anywhere between 300 days before and 300 days following estimated date of conception. Although significant coefficients (α = 0.05) were found for alternative time periods and among nonzero quartiles besides the fourth, these are dwarfed in magnitude and significance by those occurring during the first trimester of gestation among the highest quartile of exposure (Figure 2). Shifting the temporal window so that it starts just following neural tube closure (day 26) yielded the largest fourth nonzero quartile OR = 7.6 [95% confidence interval (CI), 3.1–18.6]. ORs from regression modeling using both *a priori* and *a posteriori* time periods for the organochlorine category of pesticides are presented in Table 3.

In the study area, dicofol and endosulfan accounted for > 98% (by poundage) of the organochlorines applied. During the temporal period identified through the *a posteriori* analysis (i.e., days 26–81), 88 subjects resided within 500 m of a dicofol application and 27 within 500 m of an endosulfan application. Because of these small numbers, a full set of ORs could not be calculated separately for each of the two compounds. Analysis using radii > 500 m suggested magnitudes of association slightly higher for endosulfan than for dicofol; the association of each compound with ASD appeared to be largely independent of the other (data not shown).

Our initial model controlled for maternal race and ethnicity, education, and RC of diagnosis (recorded or imputed). To assess model sensitivity, we employed the *a posteriori* time window and the 500-m buffer and investigated models using no covariates, our original covariates plus maternal age and child sex, and various combinations of these. ORs were not significantly altered under any model, although we did observe some attenuation of the association when sex was included in the model. Given the observed sex ratio among cases and our low exposure prevalence (1.5%), nearly all exposed cases were male, so this attenuation should not necessarily be construed as evidence for confounding or effect modification by sex. Inclusion of covariates besides sex nonsignificantly increased, rather than decreased, the observed association. Choice of the initial covariates plus sex in the model yielded a fourth nonzero quartile OR of 6.1 (95% CI, 2.4–15.3). Repetition of the simulated RC assignment for controls 100 times yielded a median estimate for this number of 6.1 (95% CI, 2.4–15.5), minimum 5.8 (95% CI, 2.3–14.6), and maximum 6.7 (95% CI, 2.6–17.2).

**Figure 3:**
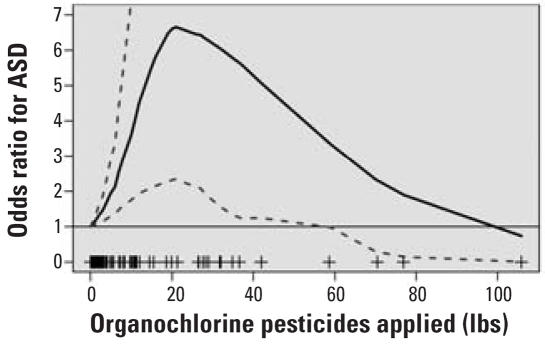
Dose–response curve for ASD risk with organochlorine pesticides applied within 500 m of residence during *a posteriori* temporal period (26–81 days postfertilization), controlling for maternal education, maternal race/ethnicity, RC of diagnosis (imputed for controls), and sex of child. Plus signs indicate data points from which curves have been calculated. Solid line, OR estimate; dotted lines, 95% confidence limits.

Characterization of the dose–response relationship between organochlorine pesticide applications and ASD risk is shown in Figure 3. Risk appears to increase monotonically up to the application amount of approximately 22 lb during the 8-week period with the highest OR determined *a posteriori*. This poundage is equivalent to the 87th percentile for the nonzero applications in the sample; beyond this magnitude of application, data are too sparse to allow for the calculation of risk, as evidenced by the widening of CIs and the attenuation of the OR back to the null.

## Discussion

The objective of this study was to systematically explore the general hypothesis that residential proximity to agricultural pesticide applications during pregnancy could be associated with ASD in offspring. By separately considering the parameters identifying *a*) compounds and compound groupings, *b*) spatial proximity, and *c*) temporal windows, we tested 249 hypotheses that met our predetermined criteria. Application of *a priori* analytic standards to reduce the statistical problems associated with testing and interpreting this large number of hypotheses led us to dismiss nearly all of these hypotheses. Statistical approaches (Aickin and Gensler 1996; Nichols and Hayasaka 2003) and visual inspection concurred that the association between organochlorine pesticide applications immediately before and during the period of CNS embryogenesis and ASD risk merited further investigation. This association was strongest for residences closest to pesticide applications and was attenuated with increasing distance. *A posteriori* analysis indicated that the association was strongest, in these data, among those residing near the highest nonzero quartile of pesticide applied during the 8 weeks immediately following cranial neural tube closure. The magnitude of this association was substantially larger than any we could generate through additional testing using alternative time periods and/or quartiles of pesticide applications. We adopted as an *a posteriori* hypothesis that these 8 weeks reflect the period of actual maximum embryonic vulnerability to the organochlorine pesticides. Findings were insensitive to choices of covariates available for our exploratory model, although the inclusion of sex as a covariate attenuated the association slightly. Within the limits of our data, ASD risk increased monotonically with the amount of organochlorine applications during this *a posteriori* time period.

### Organochlorine pesticides

Organochlorines include a chemically diverse group of halobenzene-derivative compounds used mostly as insecticides; in the study area, nearly all of the pesticide applied in this class was dicofol or endosulfan, both of which were used on cotton, fruits, vegetables, beans, and nuts.

In general, halobenzene derivatives are metabolized through the cytochrome P450 system in humans (Rietjens et al. 1997). Dicofol is chemically similar to dichlorodiphenyltrichloroethane (DDT), the difference being that dicofol possesses a hydroxy moiety on one of its two aliphatic carbon atoms. Dicofol is not metabolized to dichlorodiphenyldichloroethylene (DDE), is cleared from the body more quickly, and bioaccumulates less than DDT (U.S. Environmental Protection Agency 1998). Following oral dosage in studies with rats and mice, peak serum concentrations are reached within 24–48 hr, with most of the compound cleared from the body within 8 days (EXTOXNET 1996a). Environmentally, dicofol’s geographic and temporal distribution follows the patterns of its application because of its relative solubility, generally being detectable in field runoff only during seasons of field applications (Domagalski 1996).

In rats, endosulfan is converted by the liver after oral administration to endosulfan sulfate and endosulfan diol; peak serum concentrations are reached within hours and elimination achieved within days (Agency for Toxic Substances and Disease Registry 2000; Chan and Mohd 2005; Chan et al. 2005). In humans, the diol compound in particular has been detected in both placenta and neonatal cord blood (Cerillo et al. 2005). Less soluble than dicofol, endosulfan breaks down in soil and water over periods of weeks to months (EXTOXNET 1996b).

#### Biological activities in humans

Generally speaking, the brain has not been highlighted as the primary target organ for the toxicity of either dicofol or endosulfan. The latter compound has been noted to have estrogenic effects as well as some effects on the thyroid gland (Schantz and Widholm 2001; Soto et al. 1994), which may be relevant to concerns about the role of the fetal hormonal milieu in ASD pathogenesis (Baron-Cohen et al. 2005). Sexual differentiation of brain structures in higher mammals occurs during the fetal period (weeks 9–38) (Tsuruo 2005), and aromatase, the enzyme that converts androgens into estrogen, is expressed by nerve cells localized in specific brain structures during this period.

Both dicofol and endosulfan noncompetitively bind gamma amino-butyric acid (GABA) receptor–mediated chloride ion channels in nerve cells (Sunol et al. 1998). GABA-mediated neurotransmission is known to play important roles in gestational brain development, and the theory that altered GABA metabolism could play a role in ASD has been advanced (Cohen 2001). GABA is a neurotransmitter largely unique to interneurons, and GABA-mediated activity regulates cell migration, proliferation, synaptogenesis, and, by extension, the overall patterning of neural networks (Herlenius and Lagercrantz 2004). Mice with abnormal genes for glutamic acid decarboxylase, which is essential for GABA synthesis, develop epilepsy, abnormal neural activity, and increased anxiety-like behavior. Different forms of this enzyme appear sequentially throughout development, which is thought to imply the existence of multiple distinct functions for GABA as a neurotransmitter during different periods (Varju et al. 2001).

#### Implications for public health

Because this is the first study to explore whether risk of ASD is associated with residential exposure to organochlorine pesticides at drift concentrations, our results require replication in further studies and should be treated with caution. In particular, we want to draw the reader’s attention to the small numbers of subjects classified as “exposed” under our model that generated the largest magnitude of ASD risk (a 500-m distance between field and residence and the *a posteriori* temporal window). Using this model, a total of 113 case and control subjects were connected with pesticide applications within the spatial–temporal window, with 29 subjects (8 cases) in the fourth nonzero quartile of exposure.

Among control subjects, the prevalence of our *a posteriori*–defined exposure was 14.3/1,000 births in the Central Valley region. Assuming a baseline risk for ASD of 6.5/1,000 births (Bertrand et al. 2001; Chakrabarti and Fombonne 2001), the OR of 6.1 suggests a putative population attributable risk on the order of 7% for births to Central Valley residents. This calculation assumes that the relationship between exposure and outcome is causal and considers only exposure to drift from agricultural applications. Associations of ASD with exposure from other sources could not be considered using the present study design.

To our knowledge, neither dicofol nor endosulfan are used in household products or are used in any quantities outside of the commercial agricultural setting. Residues of both compounds are commonly detected in a wide variety of foods, however (Groth et al. 2000), as are those of persistent organochlorine compounds no longer in use as pesticides (Schafer and Kegley 2002). Both compounds have structural similarities with relatively common toxic contaminants such as hexachlorobenzene and polychlorinated biphenyls. Most chlorinated aromatic ring compounds are substantially more persistent in the environment and in human tissues than dicofol and endosulfan. The ability to detect associations between putative pesticide exposure due to agricultural drift and ASD risk in the present analysis may have been augmented by the presence of the compounds in relatively defined spatial and temporal windows.

The availability of pesticide application data in California has provided an opportunity to detect a possible link, but replication of our results and further evaluation in laboratory studies are essential to determine whether these compounds could be etiologically related to the occurrence of ASDs in some children. In this context, it may be relevant to note that the total applied poundage of endosulfan and dicofol decreased in California by approximately one-half between 1998 and 1999 but appears to have remained steady since that time (Pesticide Action Network of North America 2006).

### Study strengths and limitations

One strength of this study was the ability to locate pesticide applications with relatively high resolution in both space and time, which allowed us to operationalize hypotheses referring to specific temporal periods of vulnerability. We were able to use this and the large number of compounds for which we had data to characterize many associations, identifying those likely to arise through multiple testing and contrasting those with associations that appeared more compelling. We were able to meet many standards set for the epidemiologic study of neurodevelopmental effects of *in utero* chemical exposure (Amler et al. 2006), particularly with regard to the methodical definition and testing of plausible hypotheses *a priori*. Another strength is that prior studies have demonstrated good diagnostic validity for children reported by DDS to have autism when electronic statewide records are compared with data in RC records (Grether J, personal communication) or results of standardized evaluations conducted for specific studies (e.g., using the Autism Diagnostic Interview–Revised and the Autism Diagnostic Observation Scale) (Hertz-Picciotto et al. 2006).

Misclassification of exposure is the primary limitation of this study. It has been estimated that one in three women change addresses during pregnancy (Canfield et al. 2006). Furthermore, we were unable to assess time spent at home during the time periods in question or the influence of wind speed and direction on drift. Although it is impossible to assess, it is likely that this misclassification is nonsystematic in nature; further, the specificity of our exposure metric is likely to decrease as larger buffer distances between fields and residences are employed.

Little information was available to us describing the mothers and children in the sample other than basic demographic characteristics, so we were unable to adjust for confounders potentially important to gestational neurodevelopment, such as the use of prenatal vitamins (Shaw et al. 1995). Anecdotal evidence suggests that mothers from a wide variety of socioeconomic backgrounds and occupations were represented in the “exposed” categories, but we cannot dismiss the possibility that these women may be disproportionately employed in agriculture and therefore subject to occupational exposures to pesticides beyond drift concentrations.

ASD is relatively rare, and more mildly affected children may be less represented in our case group. The proportion of mothers in the sample living in proximity to pesticide applications during our specific time periods of interest was small. This limited the numbers of people classified as exposed at any particular level of pesticide compounds. As mentioned above, for example, only 29 subjects were classified in the fourth nonzero quartile of exposure to organochlorines using our *a posteriori* parameters. Of these, eight subjects had ASD; although this is significantly greater than the expected number (1.8), the need for replication of these findings in other, larger populations is clear.

## Conclusions

We evaluated the overarching hypothesis that maternal residence near agricultural pesticide applications in California’s Central Valley during defined time periods of gestation could be associated with ASD among children. We employed a staged analytic strategy designed to exclude associations due to multiple testing using *a priori* criteria. Risk for ASD was consistently associated with residential proximity to organochlorine pesticide applications occurring around the period of CNS embryogenesis; this association appeared to increase with dose and was attenuated with increasing distance of residence from the field site. These findings suggest that the possibility of a connection between gestational exposure to organochlorine pesticides and ASD requires further study.

## Figures and Tables

**Table 1:** Demographic characteristics of ASD cases and controls born in California Central Valley counties, 1996–1998.

Variable	Percent of cases (*n* = 465)	Percent of controls (*n* = 6,975)	Chi-square *p*-value
Sex of child			< 0.0001
Male	85.2	51.4	
Female	14.8	48.6	
Maternal age (years)			< 0.0001
< 20	8.4	14.5	
20–24	24.1	26.6	
25–29	25.6	27.2	
30–34	23.4	20.2	
35–39	15.9	9.3	
≥40	2.6	2.3	
Maternal race/ethnicity			0.01
Non-Hispanic white	50.8	44.1	
Non-Hispanic black	7.1	6.0	
Native American	0.4	0.9	
Asian	9.7	8.7	
Hispanic	31.2	39.6	
Other^a^	0.9	0.7	
Maternal education			< 0.0001
Elementary	4.3	13.0	
Some high school	12.5	19.1	
High school diploma	33.1	31.6	
Some college	28.2	21.8	
College degree	20.4	13.3	
RC of diagnosis (imputed if control)			0.07
361/Golden Gate	0.9	0.5	
362/San Diego	0.9	0.7	
363/Far Northern	6.5	7.6	
364/Alta California	25.8	25.8	
365/San Andreas	0.2	0.3	
367/Central Valley	16.3	21.2	
369/Inland	0.2	0.4	
371/North Bay	8.4	5.2	
372/Kern	12.5	11.7	
374/South Central LA	0.4	0.3	
375/Harbor	1.1	0.5	
377/Valley Mountain	25.2	23.7	
999/Other^b^	1.7	2.2	

a Includes Pacific Islander and those recorded as other.

b Includes 360/Lanterman, 366/Tri-Counties, 370/Redwood Coast, 376/Westside, 378/North LA County, 379/San Gabriel/Pomona, 368/Orange County, and 380/East Bay.

**Table 2:** Coefficients for ASD risk comparing the fourth nonzero quartile of exposure to no exposure^a^ among children born in selected California counties, 1996–1998.^b^

Temporal window	Buffer radius (m)	Coefficient	*p*-Value	Adjusted alpha^c^
Bifenthrin				
Gestation	250	1.570	0.0485	0.0047
Organochlorines				
CNS	250	2.068	0.0011*	0.0500
CNS	500	1.452	0.0025*	0.0253
CNS	750	1.178	0.0062*	0.0170
CNS	1,000	1.031	0.0064*	0.0127
Gestation	500	0.692	0.0249	0.0085
Organophosphates				
Gestation	250	0.462	0.0418	0.0057
Trifluralin				
Gestation	750	−0.839	0.0459	0.0051

Only coefficients for which a minimum of 20 subjects had nonzero exposure and *p* ≤ 0.05 are shown.

a Controlling for maternal education, maternal race/ethnicity, and RC of diagnosis (imputed for controls).

b Four hundred sixty-five cases and 6,975 controls matched by LMP date, analyzed by conditional logistic regression.

c Using the Holm algorithm and the formula of Dunn-Sidák (see “*A priori* analysis” in “Results”).

* Indicates *p* ≤ adjusted alpha.

**Table 3:** Adjusted ORs^a^ (95% CIs) for ASD among children born in selected California counties during 1996–1998, by nonzero quartile of organochlorine pesticides applied within 500 m of residence during various periods of gestation.^b^

	Neural tube (4 days pre- to 24 days postfertilization)	CNS (7 days pre- to 49 days postfertilization)	Gestation (14 days pre- fertilization to DOB)	*A posteriori* (26–81 days postfertilization)
Nonzero quartile^c^ of pounds applied (reference = 0)				
First	1.0 (0.1–7.8)	0.6 (0.1–4.3)	1.2 (0.6–2.5)	0.6 (0.1–4.3)
Second	1.2 (0.2–9.9)	1.6 (0.4–7.1)	0.8 (0.3–1.9)	0.8 (0.1–6.3)
Third	2.6 (0.6–11.9)	2.4 (0.7–8.2)	1.0 (0.5–2.2)	2.1 (0.6–7.3)
Fourth	3.5 (1.0–12.5)	4.2 (1.7–10.9)*	1.8 (1.0–3.3)	7.6 (3.1–18.6)*

DOB, date of birth.

a Adjusted for maternal education, maternal race/ethnicity, and RC of diagnosis (imputed if control).

b 465 cases and 6,975 controls matched by LMP date, analyzed by conditional logistic regression.

c 25th, 50th, and 75th percentile cut points (in pounds) for neural tube period were 0.1, 1.6, and 5.2; for CNS, 0.3, 1.9, and 8.4; for gestation, 0.3, 2.9, and 12.0; for *a posteriori* 0.3, 1.8, 10.1, respectively.

* *p* ≤ 0.05;

## Appendix 1. Pesticide compounds and categories of compounds selected for hypothesis testing

The following pesticide compounds and categories of compounds were selected from California DPR Pesticide Use Reports (2000) for hypothesis testing. Compounds in a single functional or chemical category were grouped together for hypothesis testing purposes; individual compounds were considered on their own regardless of whether they previously had been included in a group category.

**Table:**

***Functional and chemical categories.***^a^ Cholinesterase inhibitors (149 entries, including phosmet, malathion, ethephon, and thiobencarb) Copper-containing compounds (45 entries, including hydroxides, sulfates, ammonium carbonates, and oxide) Fumigants (37 entries, including chloropicrin, methyl bromide, 1,3-dichloropropene, metam-sodium, metam-potassium, and sulfuryl fluoride) Avermectins (4 entries, including avermectin, emamectin, benzoate, and fipronil) Halogenated organics^b^ (43 entries, including methyl bromide and 1,3-dichloropropene) *N*-methyl carbamates (29 entries, including carbamyl, thiodicarb, carbofuran, methomyl, and aldicarb) Organochlorines^b^ (40 entries, including dicofol, endosulfan, and dienochlor) Organophosphates (112 entries, including malathion, phosmet, chlorpyrifos, and ethephon) Pyrethroids (35 entries, including permethrin, cypermethrin, fenvalerate, cyfluthrin, esfenvalerate, and bifenthrin) Thiocarbamates [12 entries, including molinate, thiobencarb, EPTC (ethyl dipropylthiocarbamate), and pebulate]		
***Individual compounds.***
1,3-Dichloropropene	Bromacil acid	Bifenthrin
Chloropicrin	Chlorpyrifos	Copper sulfates
Cypermethrin	Dazomet	Diuron
Fenarimol	Glyphosate	Metam-sodium
Methyl bromide	Molinate	Myclobutanil
Norflurazon	Oxadiazon	Paraquat
Trifluralin		

a Categories may overlap and/or include compounds also tested individually.

b Small chlorinated molecules (e.g., 1,3-dichloropropene) and chlorinated benzenes are included as halogenated organics; polycyclic chlorinated compounds are included as organochlorines.
